# Association of chest computed tomography severity score at ICU admission and respiratory outcomes in critically ill COVID-19 patients

**DOI:** 10.1371/journal.pone.0299390

**Published:** 2024-05-02

**Authors:** Ricardo Esper Treml, Tulio Caldonazo, Fábio Barlem Hohmann, Daniel Lima da Rocha, Pedro Hilton A. Filho, Andréia L. Mori, André S. Carvalho, Juliana S. F. Serrano, Pedro A. T. Dall-Aglio, Peter Radermacher, João M. Silva

**Affiliations:** 1 Department of Anesthesiology and Intensive Care Medicine, Friedrich-Schiller-University, Jena, Germany; 2 Department of Anesthesiology, University of São Paulo, São Paulo, Brazil; 3 Department of Cardiothoracic Surgery, Friedrich-Schiller-University, Jena, Germany; 4 Department of Intensive Care Medicine, Hospital Israelita Albert Einstein, São Paulo, Brazil; 5 Department of Anesthesiology, Servidor Público Estadual Hospital, Sao Paulo, Brazil; 6 Institute for Anesthesiological Pathophysiology and Process Development, Ulm University Hospital, Ulm, Germany; Kaohsuing Medical University Hospital, TAIWAN

## Abstract

**Objective:**

To evaluate the association of a validated chest computed tomography (Chest-CT) severity score in COVID-19 patients with their respiratory outcome in the Intensive Care Unit.

**Methods:**

A single-center, prospective study evaluated patients with positive RT-PCR for COVID-19, who underwent Chest-CT and had a final COVID-19 clinical diagnosis needing invasive mechanical ventilation in the ICU. The admission chest-CT was evaluated according to a validated Chest-CT Severity Score in COVID-19 (Chest-CTSS) divided into low ≤50% (<14 points) and >50% high (≥14 points) lung parenchyma involvement. The association between the initial score and their pulmonary clinical outcomes was evaluated.

**Results:**

121 patients were clustered into the > 50% lung involvement group and 105 patients into the ≤ 50% lung involvement group. Patients ≤ 50% lung involvement (<14 points) group presented lower PEEP levels and FiO_2_ values, respectively GEE *P* = 0.09 and *P* = 0.04. The adjusted COX model found higher hazard to stay longer on invasive mechanical ventilation HR: 1.69, 95% CI, 1.02–2.80, *P* = 0.042 and the adjusted logistic regression model showed increased risk ventilator-associated pneumonia OR = 1.85 95% CI 1.01–3.39 for COVID-19 patients with > 50% lung involvement (≥14 points) on Chest-CT at ICU admission.

**Conclusion:**

COVID-19 patients with >50% lung involvement on Chest-CT admission presented higher chances to stay longer on invasive mechanical ventilation and more chances to developed ventilator-associated pneumonia.

## Introduction

Severe coronavirus disease 19 (severe COVID-19) has brought many challenges to Intensive Care Units (ICU) since December 2019 [1–3]. Chest computed tomography imaging (Chest-CT) played an important role not only in the correct early diagnosis of the infection of severe acute respiratory syndrome coronavirus type 2 (SARS-CoV-2) but also served as a pivotal tool to screening of disease severity in COVID-19 patients during the pandemic [4, 5].

Chest-CT scans of patients with COVID-19 commonly show ground-glass lesions, consolidations or mosaic-type lesions [6–8] predominantly distributed in basal and peripheral regions with predominant bilateral involvement [9]. Different methods of Chest-CT scan for COVID-19 evaluation have been proposed to improve the sensitivity and specificity of its diagnosis and to predict clinical progress, especially ICU admission [10–12] and some studies have demonstrated an association with clinical outcome using different non-validated semi-quantitative Chest-CT methods in ward patients [13–16]. A new validated COVID-19 Chest-CT Severity Score (Chest-CTSS) system based on pulmonary parenchyma involvement has been tested in ward patients and showed to be associated with clinical worsening in patients who scores were considered high (≥14 points) [17, 18].

For instance, the role of the new proposed Chest-CT severity score system in critically ill patients with COVID-19 on their pulmonary outcomes remains unclear. This prospective, single-center, observational study aims to evaluate the association between admission Chest-CTSS in patients with COVID-19 with their pulmonary outcomes.

## Materials and methods

### Study design

This is a single-center cohort prospective observational study in COVID-19 patients conducted in the ICU of a tertiary hospital in Brazil, between March 2021 to December 2021. Ethical committee approval for the study protocol was obtained (ethical approval number: CAAE: 31534120.0.0000.5463. Ethical Committee, approval date). The study was conducted according to the STROBE-Guidelines for prospective observational studies [19] and respecting the Helsinki Declaration. For all included patients, informed consent was obtained from patients and their relatives or legal representatives when patients could not provide consent.

### Study population and eligibility

We screened and selected patients older than 18 years old with confirmed infection due to SARS-CoV-2 using a positive real time polymerase chain reaction (RT-PCR) and a confirmed diagnosis of COVID-19 according to WHO guidelines [20] who required ICU treatment due to COVID-19 and need for invasive mechanical ventilation 24 hours from admission. None of the patients included in this study was vaccinated against SARS-CoV-2. All patients received the standard of care according to the Surviving Sepsis Campaign Guidelines on the Management of Critical ill Adults with COVID-19 [21]. Exclusion criteria were patients who had not been tested or had no results available RT-PCR test for SARS-CoV-2; patients who had not undergone Chest-CT scan; presence of an important movement artifact on Chest-CT; absence of a definitive clinical diagnosis; final alternative clinical or serological diagnosis; and lack of clear clinical information about duration of symptoms.

### Chest computed tomography imaging and quantitative assessment of lung parenchyma involvement

After ICU admission and confirmatory PCR for SARS-CoV-2, Chest-CT was performed on the admission day in the ICU using CT scanners Somatom Definition AS+ (Siemens Healthineers Ò); Lightspeed 16 (GE Healthcare Ò). Chest-CT images obtained from scanners with 64 to 320-detector rows of were evaluated. All examinations were performed in the supine position, during maximal inspiration, with no use of contrast medium.

**Fig 1:**
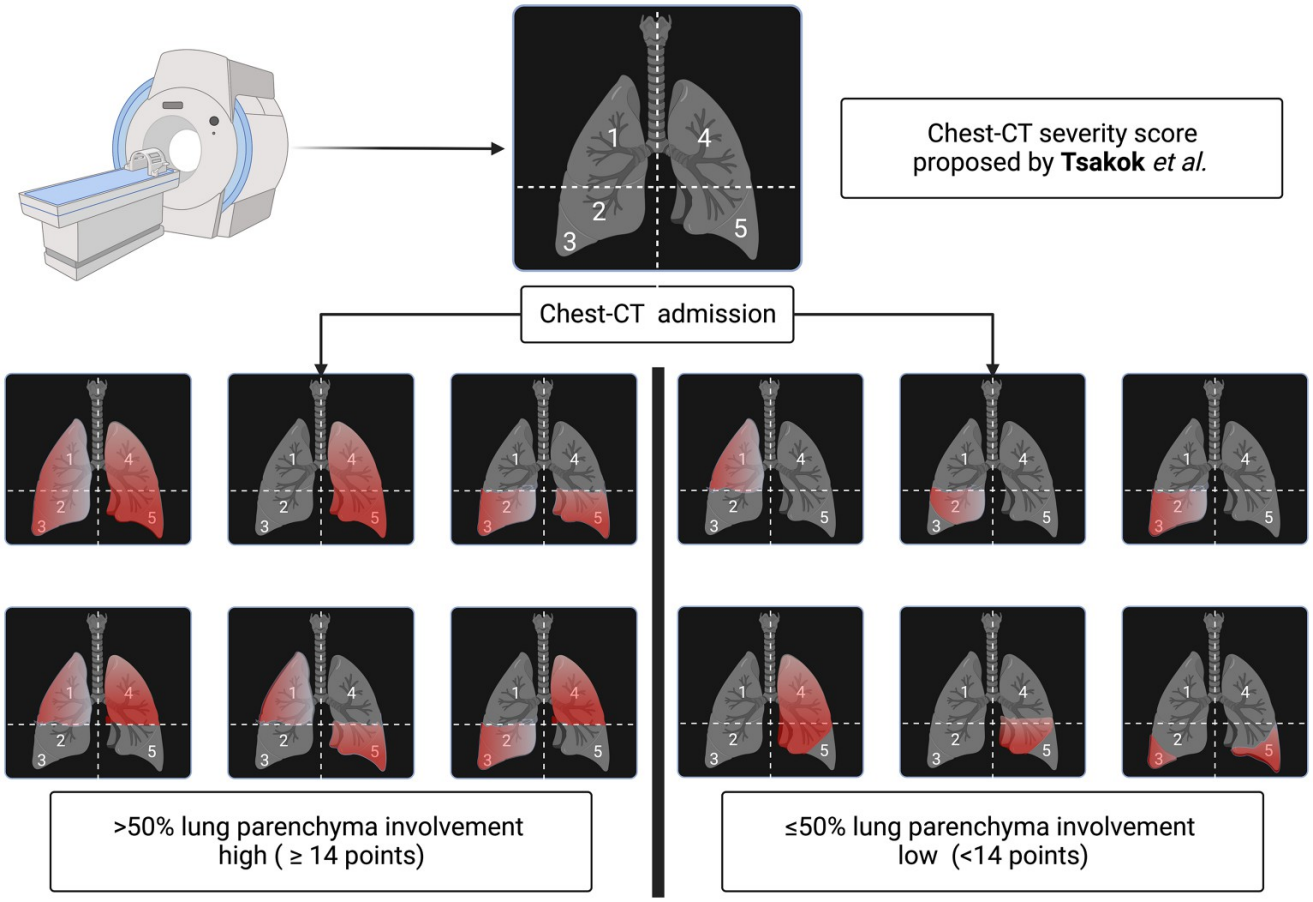
Represents the radiological classification based on lung parenchymal involvement proposed by Tsakok et al. [18]. After admission and laboratory confirmation by RT-PCR of SARS-CoV-2 infection, patients underwent Chest-CT and were classified based on theirs Chest-CT severity score in high involvement and low parenchyma involvement. Image created using BioRender.com by RET.

Image acquisition was made according to the internal protocol and international recommendations for COVID-19 imaging using a single-phase, low radiation-dose non-contrast Chest-CT at 100 kV with tin filter using voltage 80kVp to 120kVp, tube current of 10mA to 440mA [18]. Both varying according to institutional protocols already established for each device and biotype of the patient, and reconstruction with thickness ranging between 1mm and 1.5mm. The images were evaluated within a set of more than 600 cases referred to our service with suspected COVID-19 by two radiologists with two-year experience in chest imaging. Initially, the evaluators did not have access to results of RT-PCR. All patients were categorized based on Chest-CTSS proposed by Tsakok *et al*. [17, 18, 22] in semi-quantitative system divided into low ≤ 50% (<14 points) and high > 50% (≥14 points) lung parenchyma involvement (Fig 1 and S1 Fig). Disagreement cases were decided by consensus between the two radiologists.

### Primary outcomes

The primary outcomes were the risk association between score on admission Chest-CT with time on mechanical ventilator and respiratory outcomes during D_0_ (before mechanical ventilator, baseline) to 7-day in terms of ventilatory and laboratory parameters in different time points (D_0_, D_3_ and D_7_), such as, oxygenation index (PaO_2_/FiO_2_), arterial oxygen saturation (SaO_2_), positive end-expiratory pressure (PEEP), and fraction of inspired oxygen (FiO_2_).

### Secondary outcomes

As secondary outcomes, we evaluated risk association between score on admission Chest-CT and prone position rate, ventilator-associated pneumonia rate, extubating failure rate, pulmonary thromboembolism rate during D_0_ to D_28_. Further, we have exploratively evaluated the risk association on administration of vasoactive drugs at D_3_, in-hospital mortality, length of stay in the ICU and hospital (ICU-LOS and Hospital-LOSH).

### Statistical analyses

For the statistical analyses of the continuous demographic, clinical, and laboratory data, values were summarized as means (±SD) or median (Q1/3). We reported their absolute and relative absolute and relative frequencies for categorical variables. The distribution of the variables was tested using the Shapiro-Wilk test. To compare the baseline demographic D_0_ data between the groups we applied *T*-Test or Mann-Whitney U tests for the continuous variables or χ2-tests for categorical variables. For the comparison of clinical and laboratory data between groups at D_0_, D_3_ and D_7_, we used generalized estimating equations (GEE) models with marginal Poisson distribution and an identity link function, assuming a first-order autoregressive (AR1) [23] correlations between assessment times. For the direct comparison between D_0_, D_3_ and D_7_ from each group separately, we used Wilcoxon signed rank tests and the results were followed by Bonferroni multiple comparisons to identify the differences between groups and time points when significant [24, 25].

In the analyses of primary and secondary outcomes, we conducted a survival analysis using the binary results on admission Chest-CT as a dependent variable for outcomes estimating the cumulative-event probabilities. The odds ratio was calculated using a multiple logistic regression model adjusted for possible confounders (age, gender, BMI, and SAPS 3 baseline). Variables with multicollinearity were removed from the final analysis. The logistic regression models were tested using a generalized Hosmer-Lemeshow goodness-of-fit test (23). Furthermore, it was calculated adjusted (age, gender, BMI, and SAPS 3 baseline) Hazard ratio (HR) and 95% confidence between groups in relation to remaining invasive ventilation rate by length invasive mechanical ventilatory stay and after results were plotted in Kaplan-Meier curve.

Statistical analysis was performed with IBM SPSS 26 (IBM Corporation, Armonk, NY, USA) and Graphpad Prism 7.05 (Graphpad Software Inc., San Diego). We applied a significance level of 5% and reported two-sided p-values.

## Results

**Fig 2:**
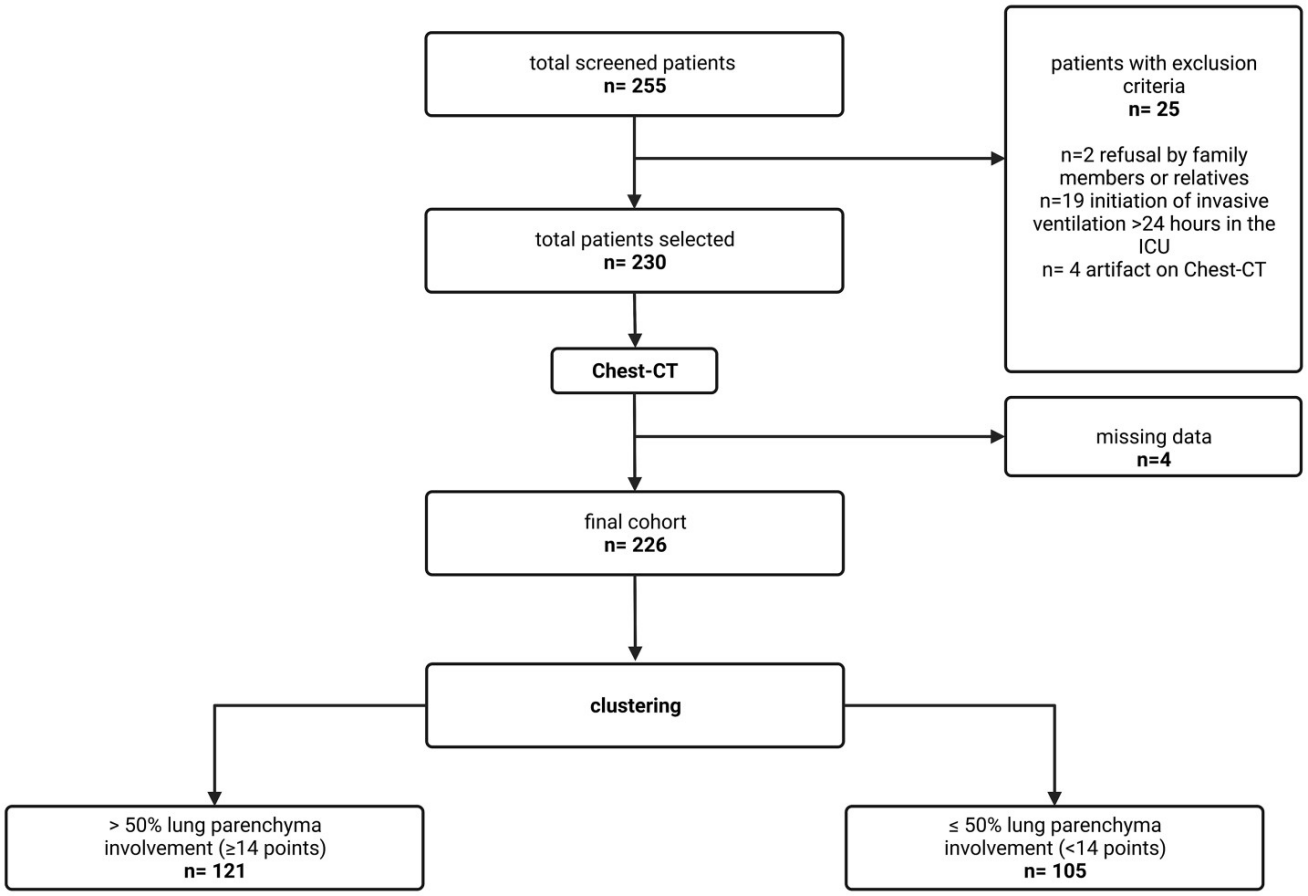
Flow diagram of study based on the assessment of chest-CT according with criterions proposed by *Tsakok et al*. The images were evaluated by two radiologists with two-year experience in chest imaging. Those patients who filled radiologic criterion for covid-19 were separated in two groups depending on their lung involvement. In the moment of the images assessment, the radiologists did not have access to results of RT-PCR.

Fig 2 describes the study design. Initially, 255 patients were screened. After removing patients based on exclusion criteria and patients who did not present complete data before D_7_, 226 patients were included in the final cohort (flowchart). They were allocated in two groups: > 50% (representing ≥ 14 points on Chest-CTSS) and ≤ 50% of lung parenchyma involvement (representing < 14 points on Chest-CTSS), so 121 patients were clustered into the first group and 105 patients into the second group.

### Baseline characteristics

Table 1 shows the descriptive demographic data. The two studied groups presented homogeneous demographic data. There was no significant difference between the groups regarding age, sex, BMI, SAPS 3 Score and ventilatory or laboratory parameters before invasive mechanical ventilation. In summary, the two cohorts were represented by patients with a median age of 68, overweight (BMI≥ 26 kg/m2), and predominantly male with comparable preexisting comorbidities and an equivalent pulmonary dysfunction.

**Table 1:** Summary of demographic and other patient data by lung involvement at clustering.

Variables	> 50% lung involvement D_0_	< 50% lung involvement D_0_	*p-value*
	(n = 121)	(n = 105)	
**Age [y]; mean±SD**	65.9±11.2	68.8±10.4	0.05
**Male sex; n [%]**	57 (54.3%)	74 (61.2%)	0.30
**Weight [kg]; mean±SD**	83.7±18.4	81.4±30.2	0.49
**Height [m]; mean±SD**	1.68±0.11	1.65±0.01	0.07
**BMI [kg/m** ^**2**^ **]; mean±SD**	29.6±7.1	28.8±12.0	0.52
**Systemic arterial hypertension; n [%]**	85 (70.8)	70 (67.3)	0.66
**Diabetes mellitus; n [%]**	53 (44.2)	45 (42.9)	0.89
**Morbid obesity; n [%]**	35 (34.7)	29 (29.3)	0.45
**Smoking; n [%]**	39 (33.1)	38 (36.5)	0.67
**Asthma; n [%]**	6 (5.0)	6 (5.8)	1.00
**Chronic kidney failure; n [%]**	18 (15.1)	22 (21.2)	0.29
**Others comorbidity; n [%]**	44 (36.4)	29 (27.6)	0.37
**baseline admission parameters (day of intubation)**			
**SAPS 3 score; mean±SD**	57.4±14.6	61.0±15.0	0.08
**PaO**_**2**_ **[mmHg]; median (Q1-Q3)**	72.6 (57.3–94.7)	72.7 (56.7–108.1)	0.21
**SaO**_**2**_ **; mean±SD**	87.5±9.1	85.7±15.5	0.29
**Respiratory rate; mean±SD**	33.9±15.7	33.6±16.2	0.88
**PaCO**_**2**_ **[mmHg]; median (Q1-Q3)**	37.3 (34–41.9)	38.1 (32.9–45.9)	0.09
**Serum bicarbonate; mean±SD**	22.7±4.1	22.8±6.3	0.88
**Creatinine [mg/dL]; median (Q1-Q3)**	1.0 (0.8–1.5)	1.04 (0.8–1.8)	0.97
**Mean arterial pressure; mean±SD**	93.1±19.8	89.7±22.5	0.25
**Lactate [mmol/L]; median (Q1-Q3)**	2.1 (1.7–2.6)	2.04 (1.7–2.9)	0.91
**Hemoglobin [g/dL]; mean±SD**	12.6±1.97	12.1±2.6	0.13

Values summarized as mean±SD and median Q1/3 (first and third quartile). p-value was calculated by the Mann-Whitney-U test for continuous variables and the #Chi-square test for categorical variables, respectively (p<0.05). BMI: body-mass-index; Others comorbidity: coronary disease, arrhythmia, heart failure, dyslipidemia, stroke, thyroid disease, previously treated cancer, and psychiatric problems; FiO_2_: Fraction of inspired oxygen; SAPS 3: Simplified Acute Physiology Score; SOFA Score: Sequential Organ Failure Assessment Score; PaO_2_: partial pressure of oxygen in the arterial blood. # Based on chest computed tomography. *Any use of dopamine, vasopressin, epinephrine, or norepinephrine for more than 1 hour.

### Clinical and laboratory parameters

Table 2 demonstrates the clinical and laboratory parameters of the studied groups at study times D_0_ and D_7_. In the ≤ 50% lung involvement group, comparing their baseline to D_7_, the analyzed values presented not a statistically significant difference regarding fluid balance and arterial serum lactate but significantly increased creatinine and a significant decrease in mean arterial pressure (MAP) and serum hemoglobin. Similarly, in > 50% lung involvement group, the comparison of the D_0_ and D_7_ values also presented no statistically significant difference in fluid balance, however, in this group, there was a significant increase in serum creatinine, arterial serum lactate and decrease in MAP and serum hemoglobin. Comparing the D_7_ data of each group, there was no statistically significant difference in variables.

**Table 2:** Clinical and laboratory data assessed at baseline (D_0_) and compared to D_7_. In both groups, it was found statistically significance between levels of creatinine, hemoglobin and mean arterial pressure observed at D_0_ and D_7_. The same difference was not found between values of fluid balance and lactate levels in both groups. When analyzed together at D7, there was not statistical difference of any analyzed data among the groups.

Variables	≤ 50% lung involvement	≤ 50% lung involvement	*P*	> 50% lung involvement	> 50% lung involvement	*p*
	D_0_ (n = 105)	D_7_ (n = 105)		D_0_ (n = 121)	D_7_ (n = 121)	
**Fluid Balance (mL)** median (Q1-Q3)	433.0 (-187 to 1236)	532.0 (203 to 1241.5)	0.25	392.0 (-50 to 1042)	407.0 (-266 to 861)	0.67
**Creatinine [mg/dL]** median (Q1-Q3)	0.9 (0.8 to 1.8)	1.0 (0.7 to 2.6)	**<0.01**	0.9 (0.8 to 1.5)	1.3 (0.8 to 2.1)	**<0.01**
**Mean arterial pressure** median (Q1-Q3)	95.5 (77 to 104)	77.0 (70.2 to 85.7)	**<0.01**	93.0 (81 to 103)	83.0 (72 to 95)	**<0.01**
**Lactate [mmol/L]** median (Q1-Q3)	2.0 (1.7 to 2.9)	2.0 (1.7 to 2.7)	0.48	2.1 (1.7 to 2.5)	2.3 (1.8 to 2.9)	**0.03**
**Hemoglobin [g/dL]** median (Q1-Q3)	13.1 (10.2 to 13.8)	10.7 (8.7 to 12.2)	**<0.001**	13.0 (11.4 to 14.0)	10.5 (9.6 to 12.1)	**<0.01**
**Comparison of D**_**7**_ ***vs*. D**_**7**_						
**Fluid Balance (mL)** median (Q1-Q3)	532.0 (203 to 1241.5)			407.0 (-266 to 861)		0.69
**Creatinine [mg/dL]** median (Q1-Q3)	1.0 (0.7 to 2.6)			1.3 (0.8 to 2.1)		0.74
**Mean arterial pressure** median (Q1-Q3)	77.0 (70.2 to 85.7)			83.0 (72 to 95)		0.16
**Lactate [mmol/L]** median (Q1-Q3)	2.0 (1.7 to 2.7)			2.3 (1.8 to 2.9)		0.27
**Hemoglobin [g/dL]** median (Q1-Q3)	10.7 (8.7 to 12.2)			10.5 (9.6 to 12.1)		0.48

Values summarized as **median** Q1/3 (first and third quartile). p-value was calculated by Wilcoxon signed rank test for the comparison between baseline(D_0_) and D_7_ continuous variables of each group. p-value<0.05.

**Fig 3:**
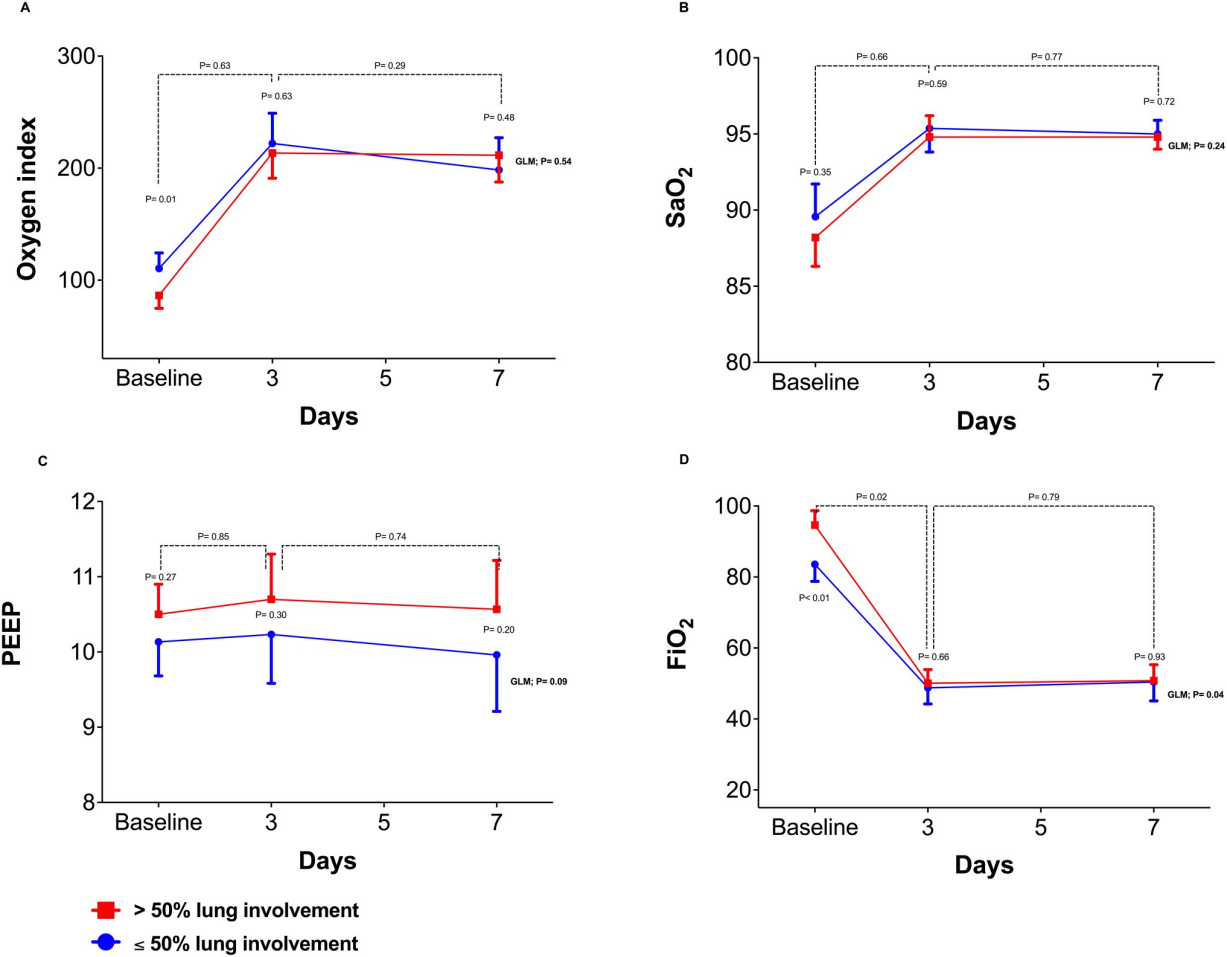
Ventilatory and laboratory parameters variation at different study time points. **(A)** Oxygen index variation from baseline by D_7_. **(B)** SaO_2_ variation from baseline by D_7_. **(C)** Adjusted PEEP levels from baseline by D_7_. **(D)** Adjusted FiO_2_ from baseline by D_7_. *SaO*_*2*_, *arterial oxygen saturation; PEEP*, *positive end-expiratory pressure; FiO*_*2*_, *fraction of inspired oxygen*.

Fig 3 shows the ventilatory and laboratory parameters at different study time points. Both groups showed a dynamic improvement in respiratory parameters such as reduced FiO_2_, increased oxygenation index, and arterial oxygen saturation. However, patients with ≤ 50% lung parenchyma involvement presented lower PEEP levels and FiO_2_ values (Fig 3C and 3D). The two groups showed similar oxygenation index and oxygen saturation with no statistical difference in all study time points (Fig 3A and 3B).

### Primary and secondary endpoints

**Fig 4:**
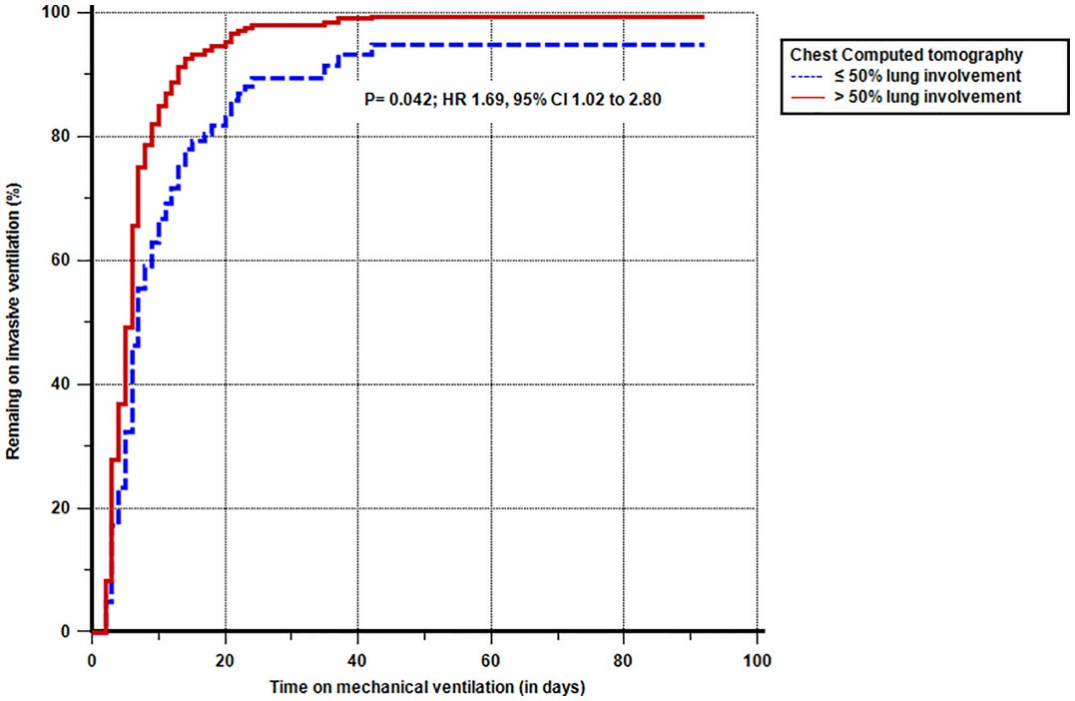
Kaplan-Meier curve plots showing the hazard ratio to remain in invasive ventilation, adjusted to minimize the effect of confounding variables.

Fig 4 shows the Kaplan-Meier remaining invasive ventilation rate curve follow-up, the adjusted to minimize the effect of confounding variables found high hazard ratio for > 50% lung involvement group HR: 1.69, 95% CI, 1.02–2.80, *P* = 0.042. Table 3 summarizes the secondary outcome. The > 50% lung involvement group showed increased risk adjusted to minimize the effect of confounding variables, of ventilator-associated pneumonia during D_0_ to D_28_ (OR = 1.85 95% CI 1.01–3.39), but all other secondaries outcomes were no statistically significant difference.

**Table 3:** Secondary outcomes summary.

Outcomes	> 50% lung involvement	≤ 50% lung involvement	Adjusted Odds Ratio		
	(n = 121)	(n = 105)	*p	odds ratio	95%CI
**Prone position from D**_**0**_ **to D**_28_*, n (%)	35(31.8)	18(23.4)	0.21	1.57	0.79 to 3.10
**Ventilator-associated pneumonia from D**_**0**_ **to D**_28_*, n (%)	47 (40.5)	25 (24.0)	<0.01	1.85	1.01 to 3.39
**Extubating Failure from D**_**0**_ **to D**_28_*, n (%)	21 (17.4)	16 (15.4)	0.69	1.16	0.55 to 2.46
**Pulmonary thromboembolism, n (%)**	17 (14.0)	12 (11.4)	0.56	1.36	0.54 to 3.47
**Administration of vasoactive drugs at D**_3_*, **n (%)**	86 (78.9)	69 (87.3)	0.13	0.55	0.22 to 1.30
Hospital mortality*, **n (%)**	73 (60.3)	79 (75.2)	0.02	0.55	0.28 to 1.09
**ICU LOS** ^#^			0.18	0.99	0.97 to 1.01
**mean±SD**	13.7±14.0	12.5±13.4			
**median (Q1-Q3)**	9 (5.7–16.2)	8.0 (3–19)			
**Hospital LOS** ^#^			0.65	0.91	0.97 to 1.00
**mean±SD**	23.5±18.5	23.3±18.0			
**median (Q1-Q3)**	19 (12–28.5)	18(10–36.2)			

D_0_: Baseline prior to initiation of invasive ventilation D_7_: 7 days after initiation of invasive ventilation; LOS: length of stay; ICU: Intensive Care Unit. The total number of patients is summarized as n, number (percentage).

^#^p-value was calculated by the Mann-Whitney U test for continuous p* value for the secondary outcomes and was estimated using multiple logistic regression adjusted for age, gender, BMI, and SAPS 3 baseline.

95%CI: confidence interval and p: p-value (<0.05 marked in bold). *The logistic regression models were tested using a generalized Hosmer-Lemeshow goodness-of-fit test (>0.05). Vasoactive drugs: *Any use of dopamine, vasopressin, epinephrine, or norepinephrine for more than 1 hour.

## Discussion

The current study showed that the validated severity score using an evaluation system based on lung parenchyma involvement on admission Chest-CT in the ICU correlated with respiratory clinical outcome in COVID-19 patients. Great lung parenchyma involvement (>50% of lung parenchyma involvement representing ≥ 14 points on Chest-CTSS) can present higher risk to remain longer on invasive mechanical ventilation and to develop more ventilator-associated pneumonia, higher FiO_2_ and PEEP requirements than those with lesser lung involvement (representing < 14 points on Chest-CTSS).

An accurate and rapid diagnosis is essential in managing cases of a highly infectious disease to offer properly allocation and medical care. The gold standard for diagnosis of COVID-19 contemplated both RT-PCR and serology. However, both tests have limitations, essentially related to the severity of symptoms and association with clinical outcome [26]. Chest-CT scan can contribute the diagnosis of COVID-19 in case of false-negative RT-PCR [27]. However, given the low-specificity tomographic patterns, the findings must be critically evaluated combined with clinical and laboratory data [28]. Moreover, in July 2020, a consensus statement on reporting Chest-CT findings related to COVID-19 was published unifying some criteria and findings standardizing the evaluation of tomographic findings in COVID-19 [29].

The evaluation of tomographic patterns in COVID-19 was also essential to predict clinical outcomes to optimizing the allocation of resources during the pandemic, a metanalysis have shown that its application in this context, using diverse methods of semi-quantitative evaluation, leads to good values of sensitivity and specificity with correlation with disease severity and mortality in COVID-19 patients [30]. However, most of this score’s systems were not validated and were not further study in a large cohort or in critically ill patients, mostly studies were involving patients on the ward [11, 12, 18, 31].

The role of the Chest-CT semi-quantitative score system to predict respiratory outcomes remains unclear in COVID-19 critically ill patients, especially those needing invasive mechanical ventilation. Thus, the present study showed, using a semi-quantitative validated Chest-CTSS, an association between parenchymal involvement and respiratory outcome. Tsakok *et al*. [18] using a modified semi-quantitative Chest-CTSS from Pan *et al*. [17] evaluated 137 patients with COVID-19 outside the intensive care setting and found an association of worse clinical outcome, representing higher chances of ICU admission and higher 30-day-mortality in patients with a scoring higher than 14 points in their semi-quantitative score system based on lung parenchymal involvement. Interestingly, in larger and different collective of patients with COVID-19, using the same semi-quantitative score we could demonstrate correlation with worse pulmonary outcomes in patients with more ≥14 points on Chest-CTSS. In our study, this group of patients had higher risk to remain longer on invasive mechanical ventilation and to develop more ventilator-associated pneumonia. Further, this group showed worse respiratory parameters as higher needs of FiO_2_ and PEEP than those with lesser lung parenchymal involvement. Our findings were comparable to the multicenter observational study *PRoVENT-COVID-19* and other studies which showed worse pulmonary parameters in critically ill patients with COVID-19 with severe lung involvement, especially in patients with more than 40% lung parenchyma involvement [3, 32, 33]. The results obtained may serve as a confirmation of the utility of Chest-CTSS) in predicting respiratory outcomes among critically ill patients with COVID-19 in the ICU setting. However, there is a compelling need for comprehensive synthesis studies and randomized trials within this specific patient cohort, utilizing the same Chest-CTSS. Given that the initial development and validation of this scoring system were conducted on non-intubated COVID-19 patients within the hospital ward, the application of a cut-off value of 14 may not be suitable for critically ill patients in the ICU. Therefore, additional research endeavors are warranted to ascertain the appropriateness of the Chest-CTSS in this distinct clinical context, ensuring its validity and accuracy for predicting respiratory outcomes in critically ill COVID-19 patients receiving intensive care.

Regarding the association of score and in-hospital mortality, we found no association between patients with higher scores and in-hospital mortality. These findings diverge from those found by a meta-analysis of several studies using different Chest-CTSS that evaluated mortality due to COVID-19 as well as other observational studies which demonstrated an increased risk of death in COVID-19 patients with high scores [34]. Some points may explain the divergent results. First, there was no standardization of the scores used in the studies [11, 12, 31, 34], generating high variance in the definitions of severity in Chest-CT. Second, the studies have mostly evaluated patients outside intensive care [11, 12, 31, 34], which makes comparison with the collective of critically ill patients difficult. Moreover, the collective of critically ill patients in our study is represented by patients with severe disease representing a higher chance of death from the disease compared to patients with mild or moderate disease, which represents more than 80% of the patients in the ward [35].

Our study has some limitations that must be considered. This was a single center prospective study, thus generalization to other centers is not recommended. Moreover, the study sample is small, not randomized and acquired data derived from a single center. The use of the proposed Chest-CTSS needs to be confirmed in larger multicenter studies in critically ill patients. Finally, the study analyzed the use of the Chest-CTSS in a group of patients with a specific disease (COVID-19) in critically ill patients, which makes difficult to reproduce these findings for other infections with interstitial infiltrative pattern inside and outside the intensive care setting.

## Conclusion

We concluded that computed tomography may have an important role in the management of cases of COVID-19 infection who present a higher lung parenchyma involvement. Patients with Chest-CTSS > 14 points showed increased risk to stay longer on invasive mechanical ventilation and to have more ventilator-associated pneumonia.

## Supporting information

S1 Fig (DOCX)

S1 Data (XLSX)
