# Supplementary material for: Association of chest computed tomography severity score at ICU admission and respiratory outcomes in critically ill COVID-19 patients
Source: PLoS One. 2024 May 2;19(5):e0299390. doi: 10.1371/journal.pone.0299390 (PMC11065208; doi:10.1371/journal.pone.0299390)

**Supplemental Digital Content**

**Supplementary Figure 1.** Cumulative fluid balance trend based on the divided groups after the model-based clustering.

**Supplementary Figure 1.**


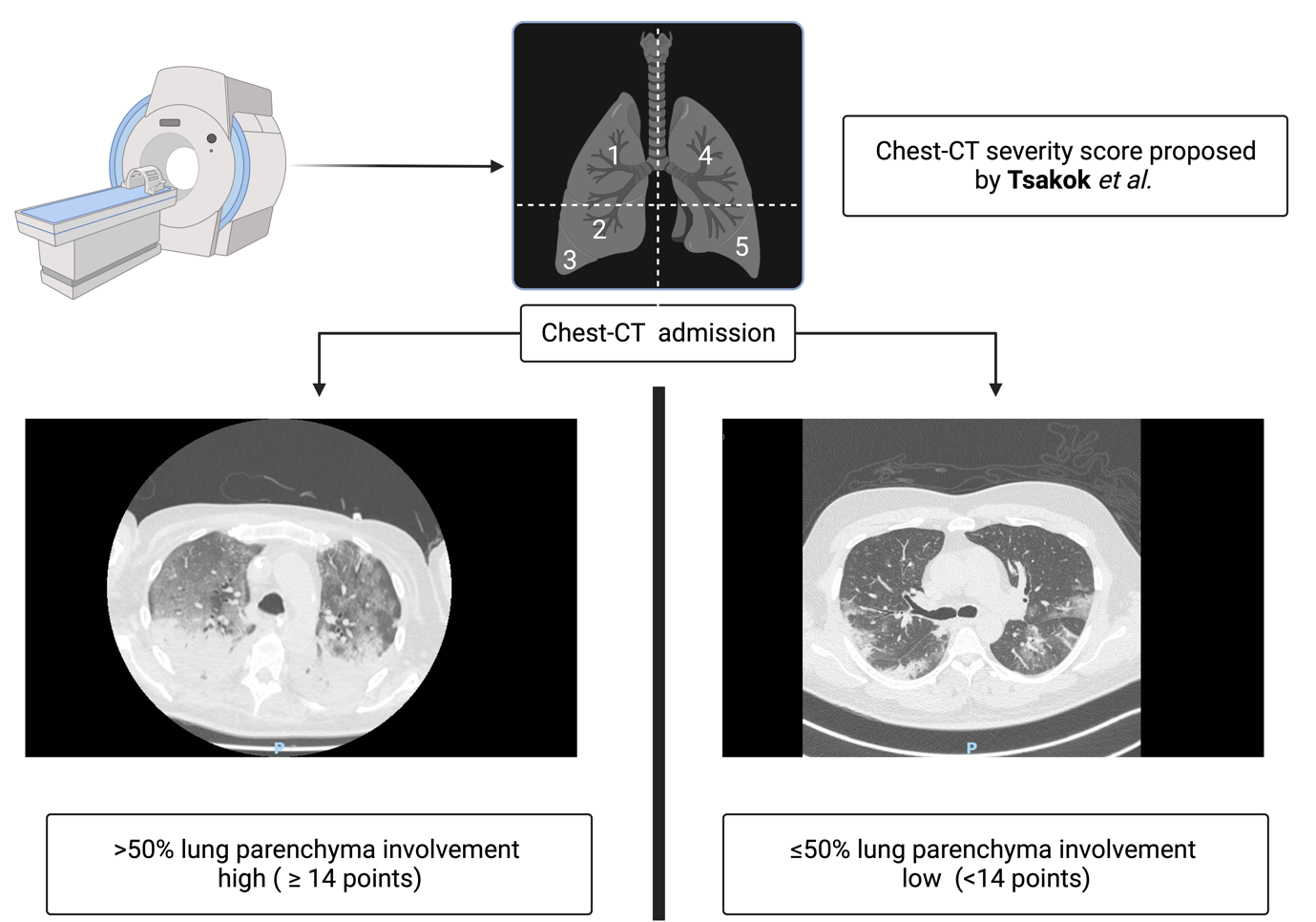

Supplement: S1 Fig — (DOCX) [file pone.0299390.s001.docx]
